# No change in key HIV target cell markers following initiation of three progestin-based hormonal contraception methods: findings from the CHIME study

**DOI:** 10.3389/fimmu.2025.1655678

**Published:** 2025-11-27

**Authors:** Marisa R. Young, C. Christina Mehta, Tyree Staple, Gina Bailey Herring, Sakthivel Govindaraj, Chris Ibegbu, Betsy C. Herold, Vijayakumar Velu, Lisa B. Haddad, Alicia K. Smith

**Affiliations:** 1 Department of Gynecology and Obstetrics, Emory University School of Medicine, Atlanta, GA, United States; 2 Division of Infectious Diseases, Department of Medicine, Emory University School of Medicine, Atlanta, GA, United States; 3 Department of Epidemiology, Rollins School of Public Health, Emory University, Atlanta, GA, United States; 4 Grady Ponce de Leon Center, Grady Health System, Atlanta, GA, United States; 5 Department of Pathology and Laboratory Medicine, Emory Vaccine Center, Emory National Primate Research Center (ENPRC), Emory University, Atlanta, GA, United States; 6 Division of Microbiology and Immunology, Emory Vaccine Center, Emory National Primate Research Center, Emory University, Atlanta, GA, United States; 7 Department of Pediatrics, Department of Microbiology and Immunology, Albert Einstein College of Medicine, New York, NY, United States; 8 Center for Biomedical Research, Population Council, New York, NY, United States

**Keywords:** mucosal immunology, CD4+ T cells, CCR5, progestin contraceptives, levonorgesterol intrauterine system, etonogestrel contraceptive implant, HIV immunology

## Abstract

**Introduction:**

Depot medroxyprogesterone acetate (DMPA) injectable, etonogestrel subdermal implant (ENG-implant), and levonorgestrel intrauterine device (LNG-IUD) are effective, widely used female hormonal contraceptives (HC). Observational studies, but not a randomized trial, suggest increased risk of HIV acquisition with HC use, particularly DMPA. Sexual acquisition of HIV occurs via CD4+ T cells expressing C-C chemokine receptor type 5 (CCR5), though other immunologic cells play a role. This study examined longitudinal changes in CCR5+ T cells and other immunologic cells in the female genital tract following HC initiation.

**Methods:**

HIV negative participants aged 18–45 years, not using HC, were recruited in Atlanta, Georgia. After two pre-HC visits, participants initiated DMPA, ENG-implant, or LNG-IUD and completed visits every three months for one year. Specimens (peripheral blood, endocervical cells, cervical tissue biopsy, and cervicovaginal lavage [CVL]) were analyzed for immune cellular markers (CD45, CD3, CD4, CCR5, CD69, HLA-DR, CD38, α4β7, CD103, Fox-P3, and Ki-67) using flow cytometry. Effects of CVL on HIV infection of cells *in vitro* was assessed. Vaginal microbiome was characterized via 16S rRNA gene amplicon sequencing. Multivariable linear mixed effects models estimated association between HC and immune markers (Primary outcome: proportion of CD4+ T cells expressing CCR5; Secondary outcomes: other immune markers, *in vitro* HIV enhancement). A Bonferroni correction was applied.

**Results:**

Among 118 participants (mean age 25.9; 44.1% self-identified as Black race), 545 visits were completed from 2019-2023. No significant changes were observed in proportion of CCR5+ T cells in any tissue type post-HC. There were statistically significant but moderate absolute decreases in proportion of CD45+ and CD4+ T cells in CVL, and CD4+ T cells in blood, and increased proportion of CD69+ T cells in blood post-HC. Post-HC CVL vs. pre-HC enhanced HIV infection of cells *in vitro* for all three HC groups (p<0.01) and was modified by the vaginal microbiome. There was also evidence of interaction by time and microbiome parameters for several other immune cells.

**Discussion:**

Our findings suggest that commonly used HC methods do not result in immunologic changes that increase HIV acquisition risk. However, HIV infection enhancement with post-HC CVL *in vitro* warrants further study.

## Introduction

Approximately 50% of reproductive-age women worldwide utilize a modern contraceptive method (1). Three highly effective progestin containing methods: the depot medroxyprogesterone acetate (DMPA) injectable, the etonogestrel subdermal implant (ENG-implant), and the levonorgestrel intrauterine device (LNG-IUD) together account for nearly a third of modern contraception utilized globally (1, 2). Access to safe and effective contraception is a public health priority. Contraception confers many benefits at the individual, familial, and societal levels including: prevention of undesired pregnancy, preventing maternal and neonatal mortality and other adverse perinatal outcomes, improved economic and educational opportunities for families, and promotion of gender equality (2). Hormonal contraception (HC) confers additional non-contraceptive medical benefits including improvement in heavy menstrual bleeding and pain, and decreased risk of ovarian and endometrial cancers (3). Despite this, there is an estimated 163 million women globally with unmet need for contraception, of whom 60% reside in sub-Saharan Africa or South Asia and one quarter of whom are aged 15-24 (1).

In the 2010s, several systematic reviews of observational studies found increased risk of HIV acquisition in women using hormonal contraception, particularly DMPA, though individual study findings were inconsistent (4, 5). Of particular concern is that confounding, or the potential that women who use contraception may have different sexual risk behaviors (e.g., reduced condom use given lack of need for pregnancy prevention) compared to non-users could explain the observed association. This is especially salient given adolescent and young women and those residing in sub-Saharan Africa and South Asia disproportionately bear the burden of both unmet contraceptive need and the HIV epidemic (1, 6).

Heterosexual HIV acquisition in women occurs almost exclusively via CD4+ T-lymphocytes expressing the cell surface receptor C-C chemokine receptor type 5 (CCR5, the primary HIV co-receptor) at the cervicovaginal epithelial barrier (7). Within the female genital tract (FGT) mucosa, the number and type of cellular targets, especially CCR5+ CD4+ T cells, is an indicator of HIV susceptibility, although these cells are heterogenous with regards to HIV susceptibility (7). Cellular characteristics, in addition to interactions with other HIV target immune cells, affect host HIV acquisition risk (8). For example, certain T cell phenotypes, such as activated T cells (e.g., cells expressing HLA-DR and CD69 markers) and cells expressing homing markers (e.g., α4β7 and CCR7) have been associated with increased risk of HIV acquisition (9, 10). It is well established that the dominant female endogenous hormones, estrogen and progesterone, can alter the innate and adaptive immune responses, thus it is mechanistically feasible for exogenous HC to also alter immune function, directly through interacting with hormone receptors and via altering endogenous hormone concentrations (9–12).

In 2019, findings from the Evidence for Contraceptive Options and HIV Outcomes (ECHO) trial were published, demonstrating no increased risk of HIV acquisition in women randomized to DMPA versus those randomized to copper IUD or LNG-implant (13). Although reassuring, the trial was not designed to identify associations with a hazard ratio below 1.5 nor to evaluate the underlying immunologic changes associated with contraception use. While the randomized design of ECHO mitigates the potential for unmeasured confounding, an updated systematic review and meta-analysis in 2020 continued to find evidence of increased HIV acquisition in DMPA users among observational studies (14). The CHIME study (Evaluating the impact of three progestin-based hormonal contraceptive methods on immunologic changes in the female genital tract and systemically) was undertaken to further mechanistic understanding of the possible link between HC use and HIV acquisition risk and add information on two contraceptives with limited available data and not included in the ECHO trial (ENG-implant and LNG-IUD). We aimed to determine the longitudinal effects of initiating three progestin based contraceptive methods (DMPA, ENG-implant, and LNG-IUD) on CCR5+ CD4+ T cells and other immunologic cells in the FGT and systemic compartments among reproductive aged women in Atlanta, GA.

## Materials and methods

### Participants

Participants were enrolled into CHIME between February 2019 and April 2023. Study participants were referred from gynecology clinics at Grady Memorial Hospital (a safety net hospital in Atlanta, Georgia), through self-referrals from fliers posted in the community and at universities, or via web-based recruitment through ResearchMatch and Trialfacts. Participants were eligible for inclusion in CHIME if they met the following criteria: assigned female at birth, younger than 45 years of age, regular menses occurring within 22–35 day intervals for at least the two previous cycles, desiring initiating DMPA, ENG-implant, or LNG-IUD, and agreeing to abstain from vaginal intercourse and use of intravaginal products for one day prior to each study visit. These three contraceptive methods were chosen as they are the only long-acting, progestin-based methods approved for use in the United States. Inclusion of long-acting methods mitigates potential concerns about user adherence that could introduce bias. Exclusion criteria included: HIV positive, pregnant or planning to become pregnant within the next year, history of a loop electrosurgical excision procedure, conization, or cryosurgery within the past year, current use of systemic HC or an IUD, or medical contraindication to the contraceptive method of choice based on the US Centers for Disease Control and Prevention Medical Eligibility Criteria (15).

Upon successful screening, visit one occurred during the luteal phase of the menstrual cycle (as determined by the onset of menses) with visit two was timed to occur within the follicular phase. Participants chose the contraceptive method, which was initiated at the completion of visit two. Following HC initiation, visits occurred at 3-month intervals through one year. Study visits included a brief questionnaire, pelvic examination, and collection of specimens. Detailed descriptions of the clinical evaluations, specimen collection methods, and laboratory assays were published in our protocol paper (16). Briefly, a trained study clinician performed the clinical evaluation and all specimen collections. A questionnaire was administered that included information on medication exposures (pain medications, probiotics, oral antibiotics, acid reducing medication, steroids, chemotherapy, antifungal medications, and antiviral medications) and medical history (tobacco use and immunocompromising conditions). During the pelvic exam, after insertion of a speculum without gel, a vaginal swab was collected for microbiome evaluation. A cervical swab was then collected for sexually transmitted infection (STI) testing (*Neisseria gonorrhoeae*, *Chlamydia trachomatis*, and *Trichomonas vaginalis*). Cervicovaginal fluid was collected by cervicovaginal lavage (CVL) according to a standardized protocol (16). Endocervical cells were then collected using a cytobrush. Finally, up to four cervical biopsies were collected (among participants consenting separately to biopsy). Peripheral venous blood was collected at each visit in cell preparation tubes and serum separator tube for immunologic markers and hormonal assessment, respectively. The study was reviewed and approved by the Emory University Institutional Review Board and the Grady Health System Research Oversight Committee.

### Sample processing and flow cytometry

As described in our prior work (12), all samples were processed within three hours of collection. CVL specimens were tested for semen exposure (Abacus ABAcard^®^ p30 test) and for qualitative presence of blood and leukocytes (Mutistix^®^ 8SG urinalysis strips). CVL samples were centrifuged to separate into supernatant and cellular fractions. Endocervical cytobrush samples were manually scraped to release cells and strained with a 100μm cell strainer then centrifuged. Cervical biopsy samples were processed using enzymatic digestion with collagenase type IV (~200 units of collagenase/ml). Prior studies in humans and primates support that collagenase digestion at this concentration does not result in the loss or downregulation of T cell markers, including CCR5 (17). All three FGT compartment samples (endocervix, CVL, and cervical tissue biopsy) were suspended in complete media, counted, and used for flow cytometry. PBMCs were isolated from whole blood collected in sodium citrate tubes and isolated by density gradient centrifugation.

As depicted in **Supplementary Figure S1**, cells were stained with fluorochrome-conjugated antibodies specific for: CD3 (SP34-2), CD4 (L200), CD8 (RPA-T8), CCR5 (3A9), HLA-DR (SK1), and Ki-67 (B56) from BD Biosciences (San Jose, CA); CD45 (HI30), CD56 (HCD56), CD38 (HIT2), CD69 (FN50), and FoxP3 (206D) from Biolegend (San Diego, CA); CD103 (HML-1) from Beckman Coulter (Brea, CA); and α4β7 (ACT-1) from the NIH Nonhuman Primate Reagent Resource. Dead cells were excluded from the analysis based on staining for Live/Dead NearIR dead cell stain from Molecular Probes, Invitrogen (Waltham, MA). Staining for Fox-P3 was performed after cells were stained for surface antigens followed by permeabilization/fixation using the Fox-P3 kit and protocol, followed by intracellular staining. Samples were acquired on an LSR Fortessa (BD Biosciences, Franklin Lakes, NJ), and all cellular events were collected for FGT samples while 500,000 events were collected for samples from blood.

### Ex vivo HIV inhibitory activity in FGT secretions

The effects of genital tract secretions on HIV infection of cells *in vitro* was assessed as previously described (18). Briefly, TZM-bl cells were infected with HIV-1BaL (~10^3^ TCID50) mixed 1:1 with CVL or control buffer (phosphate buffered saline containing 200 mg/mL bovine serum albumin) and infection monitored by quantifying luciferase activity 48 hours after infection. All samples were tested in triplicate. Values are reported as the mean percentage relative luciferase units (RLU) relative to controls; values >100% indicate enhancement and <100% indicate inhibition of HIV infection.

### Microbiome specimen processing

Vaginal swabs were collected and stored at -80°C for microbiome characterization. Microbial DNA was extracted from swabs using the DNeasy PowerSoil Kit, and the V3–V4 region of the 16S rRNA gene was amplified using the Illumina MiSeq platform and according to the manufacturer’s instructions. As we have described previously (16), QIIMEII was used for initial sample quality control followed by processing dada2 for read assembly, taxonomy assignment of amplicon sequence variants, and classification. Community state type (CST) was assigned to each sample using hierarchical clustering with the Jensen-Shannon divergence and Ward linkage (19, 20). CST-IV, characterized by lack of *Lactobacillus* predominance and comprising a diverse set of strict and facultative anaerobes, was used to operationalize molecular-bacterial vaginosis (BV) (21). We also calculated acid-producing *Lactobacillus* proportion, which excluded *Lactobacillus iners*, since this species is associated with a transitional state (22).

### Statistical analysis

Participant clinical and demographic characteristics are described by count and percent for categorical variables and mean and standard deviation for continuous variables. To account for repeated measures within a participant, mixed effect models were used and included a random intercept for participant with an unstructured covariance matrix. Models were estimated for each specimen type (blood, CVL, cytobrush, biopsy) separately. Detailed comparison of cellular markers by menstrual phase were previously published (12) and noted a small but significant decrease in CCR5+ T cells in the luteal vs. follicular phase. However, the purpose of the current analysis was to estimate the effect of progestin-based HC use on immune markers. Thus, we compared the pre-HC visits (1 and 2) to the post-HC visits (3–8) (See **Supplementary Figures S2**-**S13**). Separate models for each immunologic outcome included the following covariates: time (pre-HC: visits 1 and 2 and post-HC: visits 3-8), contraceptive method (DMPA, ENG-implant, LNG-IUD), and an HC method by time interaction term. Models with each microbial outcome (molecular-BV yes/no and *Lactobacillus* proportion ≥50%/<50%) were estimated and included the following covariates: time (pre-HC/post-HC), contraceptive method (DMPA, ENG-implant, LNG-IUD), and an HC method by time interaction term. We evaluated effect modification in the cellular immune outcomes by microbiome characteristics by modeling each immune marker outcome with the following independent variables: microbial variable (i.e., molecular-BV or acid-producing *Lactobacillus*), a combination variable of HC/visit (pre-HC, post-HC DMPA, post-HC ENG-implant, and post-HC LNG-IUD), and their interaction in mixed models with the same structure as described above. Finally, we estimated linear mixed models with the *in vitro* HIV activity outcome (percent HIV activity relative to control) and included time (pre-HC/post-HC), microbiome characteristic, and a time by microbiome interaction term. Age (continuous), race (Black/White/Other), Hispanic ethnicity (yes/no), STI presence (any *N. gonorrhoeae*, *C. trachomatis*, or *T. vaginalis* vs none), and menstrual cycle phase (follicular/luteal) were examined for association with the cellular markers. There was no difference in cellular markers based on these factors, so they were not included as covariates in the models although CVL and cytobrush models include blood contamination (yes/no) as a covariate. Models examining HIV enhancement *in vitro* include days between sample collection and assay as a covariate. Models including microbiome characteristics include race as a covariate. Model-based estimates, 95% confidence intervals, and p-values are reported.

The primary outcome of interest is the proportion of CD4+ T cells expressing CCR5. Secondary outcomes are proportion of other immunologic cellular markers and the ratio of CD4+ to CD8+ T cells from the flow cytometry that may provide deeper insight into immune regulation of T cells in the FGT. Statistical significance was set at α=0.05 for the primary outcome of CCR5-expressing CD4+ T cells. A Bonferroni correction was applied to the other 10 cellular markers such that absolute statistical significance was set at α=0.05 for the combination of these markers. Analyses were conducted using SAS v9.4 software (SAS Institute, Cary, NC) and R (R Core Team).

## Results

Overall, 118 participants were eligible for inclusion, provided informed consent, and enrolled in the study. The mean age of participants was 25.9 years (standard deviation 6.7 years). Fifty-two women (44.1%) self-identified as Black race and 19 (16.1%) as being of Hispanic ethnicity. Sixty-three participants (54.8%) reported using medication for pain or inflammation in the three months prior to study enrollment. No participant reported use of injectable steroids or chemotherapy. Fewer than 8 individuals (6.8%) reported use of any of the following: acid reducing medication, oral steroids, oral antifungals, acyclovir/valacyclovir, and/or antifungal cream. Scarcity precluded further consideration for analyses. For contraception, 19 participants (16.1%) selected DMPA, 46 (39.0%) selected ENG-implant, and 53 (44.9%) selected LNG-IUD. Women in the DMPA group were older (p<0.05) and more likely to self-report Black race (p=0.01). Those in the ENG-implant group were more likely to use pain medication (p=0.04), **Table 1**.

**Table 1:** Description of cohort characteristics overall and by contraceptive group.

Characteristic	Overall N=118 N (%)	DMPA N=19 N (%)	ENG-implant N=46 N (%)	LNG-IUD N=53 N (%)	P-value*
Race					
Black	52 (44.1)	14 (73.7)	18 (39.1)	20 (37.7)	**0.01**
White	34 (28.8)	0 (0.0)	13 (28.3)	21 (39.6)	
Other	32 (27.1)	5 (26.3)	15 (32.6)	12 (22.6)	
Hispanic ethnicity					
Yes	19 (16.1)	4 (21.1)	6 (13.0)	9 (17.0)	0.71
No	99 (83.9)	15 (79.0)	40 (87.0)	44 (83.0)	
*Age*, years (Mean ± SD)	25.9 ± 6.7	29.2 ± 8.7	24.7 ± 5.9	25.7 ± 6.4	**0.05**
*Smoked cigarettes in last 3 months*	12 (37.5)	6 (66.7)	3 (27.3)	3 (25.0)	0.12†
Type of medication use in last 3 months¶					
Pain or inflammation	63 (54.8)	9 (50.0)	35 (67.3)	19 (42.2)	**0.04**
Probiotics	13 (11.4)	2 (11.1)	5 (9.8)	6 (13.3)	0.86
Oral antibiotic or antibacterial	14 (12.2)	0 (0.0)	5 (9.6)	9 (20.0)	0.07

DMPA, depot medroxyprogesterone acetate; ENG, etonogestrel; LNG-IUD, levonorgestrel intrauterine device.

Column percents may not total 100% due to rounding.

¶Medication use not mutually exclusive.

*Chi-square or simple linear regression unless otherwise noted.

†Fisher exact test. Bold indicates p<0.05.

We conducted 545 total study visits with 313 (57.4%) occurring after contraception initiation. An STI (*N. gonorrhoeae*, *C. trachomatis*, or *T. vaginalis*) was diagnosed at 9 visits (1.7%). Moderate or large amounts of leukocytes were detected in CVL at 398 visits (73.0%). DMPA visits had higher rates of an STI diagnosis and presence of semen. The pattern of qualitative CVL leukocytes was differential across the contraceptive groups. Participants that selected the DMPA and ENG-implant had higher rates of molecular-BV relative to the participants that selected the LNG-IUD. Additional visit characteristics are listed in **Table 2**.

**Table 2:** Characteristics of study visits one through eight for cohort overall and by contraceptive group.

Characteristic	Overall N=545 N (%)	DMPA N=73 N (%)	ENG-implant N=223 N (%)	LNG-IUD N=249 N (%)	p-value
Any vaginal STI*					
Yes	9 (1.7)	5 (6.9)	1 (0.5)	3 (1.2)	<0.01**
No	536 (98.4)	68 (93.2)	222 (99.5)	246 (98.8)	
Semen present					
Positive	38 (7.0)	10 (13.7)	16 (7.2)	12 (4.9)	**0.03**
Negative	504 (93.0)	63 (86.3)	206 (92.8)	235 (95.1)	
Leukocytes					
Negative	33 (6.1)	8 (11.0)	20 (9.0)	5 (2.0)	**0.01**
Trace	33 (6.1)	7 (9.6)	7 (3.1)	19 (7.6)	
Small	81 (14.9)	10 (13.7)	35 (15.7)	36 (14.4)	
Moderate	309 (56.7)	33 (45.2)	128 (57.4)	148 (59.4)	
Large	89 (16.3)	15 (20.6)	33 (14.8)	41 (16.5)	
Visit category					
Pre-contraception	232 (42.6)	36 (49.3)	92 (41.3)	104 (41.8)	0.26
Short-term post-contraception	153 (28.1)	23 (31.5)	66 (29.6)	64 (25.7)	
Mid/long-term post-contraception	160 (29.4)	14 (19.2)	65 (29.2)	81 (32.5)	
On contraception at visit					
Yes	313 (57.4)	37 (50.7)	131 (58.7)	145 (58.2)	0.45
No	232 (42.6)	36 (49.3)	92 (41.3)	104 (41.8)	
Molecular-BV					
Yes	156 (29.7)	26 (38.2)	76 (35.4)	54 (22.3)	**<0.01**
No	369 (70.3)	42 (61.8)	139 (64.7)	188 (77.7)	
Lactobacillus ≥50%^¶^					
Yes	220 (41.30)	27 (39.7)	91 (42.3)	102 (42.1)	0.92
No	305 (58.1)	41 (60.3)	124 (57.7)	140 (57.9)	
Immunocompromising condition	9 (1.7)	0 (0.0)	3 (1.3)	6 (2.8)	0.31**

DMPA, depot medroxyprogesterone acetate; ENG, etonogestrel; LNG-IUD, levonorgestrel intrauterine device; STI, sexually transmitted infection.

*Includes *Neisseria gonorrhoeae*, *Chlamydia trachomatis*, and *Trichomonas vaginalis*.

**Fisher exact test.

¶This is % of acid-producing *Lactobacillus* (excludes *L. iners*). Bold indicates p<0.05.

After longitudinal data visualization (**Supplementary Figures S2**-**S13**) and statistical assessment of cellular markers by contraception group across time, the pre-HC visits (1 and 2) and post-HC visits (2–8) were modeled jointly. Results from random effects linear mixed regression models with HC, time, and the interaction of the two are presented in **Table 3**. There were no differences in proportion of CD45+ leukocytes, CD3+ T cells, CD4+ T cells, or CCR5+ CD4+ T cells in the endocervix or cervical tissue biopsy from pre-contraception to post-contraception initiation. In blood, there was a small decrease in the proportion of CD4+ T cells after initiation of HC, but no difference in CCR5+ CD4+ T cells or the other cellular proportions. In the CVL compartment, the proportion of CD45+ cells and CD4+ T cells decreased modestly post-contraception, but CCR5+ CD4+ T cells were unchanged (p=0.76). There was a significant interaction (p<0.01) between HC and time observed in CVL for CCR5+ CD4+ T cells, which was explored in separate linear mixed regression models for each tissue type that included HC method (DMPA, ENG-implant, LNG-IUD), time (pre/post HC), and the interaction of method and time (**Supplementary Table S1**). The CCR5+ stratum-specific cellular proportion for each contraceptive method before and after HC revealed no difference in CCR5+ CD4+ T cells between pre-HC and post-HC for DMPA or ENG-implant for any tissue type (endocervix, cervical tissue, CVL, blood). There was a significant decrease in the proportion of CCR5+ CD4+ T cells in CVL among LNG-IUD users: pre-HC 40.6 (95% CI 36.1, 45.1) to post-HC 32.4 (95% CI 27.9, 36.9; p<0.01). See **Supplementary Table S1**.

**Table 3:** Change in proportion of HIV target cells across the lower genital tract and blood of adult HIV-negative women initiating progestin-based contraception.

Tissue	Marker	Pre-HC^¶^ Estimate (95% CI)	Post-HC^†^ Estimate (95% CI)	p-value*	p-value** (HC*time)
Endocervical cells	CD45	45.9 (41.9-50.0)	44.4 (40.0-48.8)	0.55	0.96
	CD3	53.8 (50.5- 57.2)	55.1(51.4- 58.7)	0.52	0.12
	CD4	54.8 (52.9- 56.7)	52.9 (50.9-55.0)	0.09	0.36
	CCR5	39.3 (36.4-42.3)	41.0 (37.8-44.1)	0.34	0.26
Cervical tissue	CD45	60.6 (54.4-66.8)	55.6 (48.6-62.6)	0.27	0.90
	CD3	73.7 (70.8-76.6)	74.0 (70.8-77.2)	0.89	0.38
	CD4	53.5 (50.3-56.6)	50.8 (47.1-54.5)	0.20	0.12
	CCR5	49.8 (45.1-54.6)	49.6 (44.2-55.1)	0.96	0.11
CVL	CD45	18.2 (15.2-21.2)	14.3 (11.1-17.6)	**0.02**	0.19
	CD3	30.8 (27.0-34.6)	32.6 (28.5-36.7)	0.43	0.20
	CD4	48.6 (45.6-51.6)	44.2 (41.0-47.4)	**0.02**	0.38
	CCR5	37.7 (34.3-41.1)	37.1 (33.4-40.7)	0.76	**0.01**
Blood	CD45	94.5 (93.1-95.9)	94.8 (93.4-96.2)	0.78	0.51
	CD3	73.1 (71.6-74.5)	73.4 (71.9-74.9)	0.65	0.18
	CD4	60.1 (58.1-62.1)	58.2 (56.1-60.2)	**0.03**	0.66
	CCR5	7.0 (6.0-7.9)	7.8 (6.9-8.8)	0.17	0.27

CVL, cervicovaginal lavage; HC, hormonal contraception.

¶Includes visits 1 and 2 (see Methods).

†Includes visits 3-8 (see Methods).

*Compares cellular markers across all HC methods.

**Examines the interaction between each HC method and time. Bold indicates p<0.05.

Secondary outcomes included cellular markers for T cell activation (HLA-DR, CD69, CD38), trafficking (CD103, α4β7), regulation (Fox-P3), proliferation (Ki-67), and tissue residency (CD69, CD103) and the ratio of CD4+ to CD8+ T cells. We estimated multivariable linear mixed effects regression models with the outcome of cellular marker and the independent variables HC, time, and the interaction of HC and time (**Table 4**). There were no differences between pre-HC and post-HC in any of the four compartments (endocervix, cervical tissue, CVL, or blood) except for a small increase in the proportion of CD69+ cells in blood after HC initiation (from 1.2 to 2.1, p=0.01) and a decrease in the CD4:CD8 ratio in cervical tissue and CVL after HC initiation (p=0.01 for both).

**Table 4:** Change in CD4+ T cell activation (HLA-DR, CD69, CD38), cell trafficking (CD103, α4β7), cell regulation (Fox-P3), proliferation (Ki67), and tissue residence (CD69, CD103), and ratio of CD4+ to CD8+ across the lower genital tract and blood of adult HIV-negative women initiating progestin-based contraception.

Tissue	Marker	Pre-HC^¶^ Estimate (95% CI)	Post-HC^†^ Estimate (95% CI)	p-value*	p-value** (HC*time)
Endocervical cells	CD69	29.9 (26.9-32.9)	33.1 (29.9-36.3)	0.09	0.70
	HLA-DR	4.8 (4.0-5.7)	4.9 (3.9-5.8)	0.96	0.51
	CD38	38.8 (36.6-40.9)	40.7 (38.4-43.0)	0.16	0.69
	α4β7	58.2 (55.8-60.7)	59.0 (56.4-61.6)	0.54	0.39
	CD103	12.9 (11.5-14.3)	12.7 (11.2-14.2)	0.80	0.72
	Fox-P3	9.2 (8.1-10.4)	9.8 (8.5-11.0)	0.48	**0.04**
	Ki-67	8.7 (7.4-9.9)	9.1 (7.8-10.4)	0.61	**0.04**
	CD4:CD8	2.1 (1.8-2.4)	2.0 (1.7-2.3)	0.62	0.21
Cervical tissue	CD69	51.4 (46.0-56.8)	53.9 (47.7-60.0)	0.53	0.18
	HLA-DR	5.2 (2.8-7.6)	7.3 (4.7-9.9)	0.24	0.62
	CD38	39.7 (35.9-43.5)	40.1 (35.9-44.4)	0.88	0.45
	α4β7	58.7 (54.8-62.6)	57.6 (53.1-62.1)	0.70	0.95
	CD103	15.4 (11.9-18.9)	17.5 (13.5-21.5)	0.41	0.36
	Fox-P3	7.7 (6.3-9.1)	6.6 (5.1-8.2)	0.30	0.63
	Ki-67	4.8 (3.9-5.8)	4.3 (3.3-5.4)	0.48	0.24
	CD4:CD8	1.7 (1.5-1.9)	1.4 (1.2-1.6)	**0.01**	0.08
CVL	CD69	31.2 (27.8-34.7)	31.2 (27.4-34.9)	0.97	0.08
	HLA-DR	11.1 (9.0-13.2)	11.9 (9.7-14.2)	0.51	0.76
	CD38	42.1 (39.3-44.8)	45.4 (42.5-48.4)	0.06	0.55
	α4β7	57.7 (55.0-60.3)	56.9 (54.0-59.8)	0.63	0.61
	CD103	19.4 (16.6-22.2)	18.4 (15.4-21.4)	0.56	0.41
	Fox-P3	12.8 (10.9-14.6)	13.5 (11.6-15.4)	0.54	**0.01**
	Ki-67	9.5 (8.2-10.8)	9.7 (8.3-11.0)	0.84	0.52
	CD4:CD8	4.4 (3.1-5.7)	2.0 (0.7-3.3)	**0.01**	**0.04**
Blood	CD69	1.2 (0.7-1.7)	2.1 (1.6-2.5)	**0.01**	0.07
	HLA-DR	2.4 (2.0-2.7)	2.5 (2.1-2.9)	0.61	0.72
	CD38	58.5 (56.7-60.2)	60.2 (58.4-62.0)	0.09	0.32
	α4β7	64.0 (61.9-66.0)	64.4 (62.3-66.5)	0.69	0.56
	CD103	1.2 (1.1-1.3)	1.3 (1.2-1.4)	0.18	0.31
	Fox-P3	2.2 (1.9-2.6)	2.1 (1.7-2.4)	0.45	0.38
	Ki-67	2.0 (1.7-2.3)	2.3 (2.0-2.6)	0.20	0.14
	CD4:CD8	2.3 (1.9-2.7)	2.2 (1.8-2.6)	0.77	0.93

CVL, cervicovaginal lavage; HC, hormonal contraception.

¶Includes visits 1 and 2 (see Methods).

†Includes visits 3-8 (see Methods).

*Compares cellular markers across all HC methods.

**Examines the interaction between different HC methods and time. Bold indicates p<0.05.

**Figure 1:**
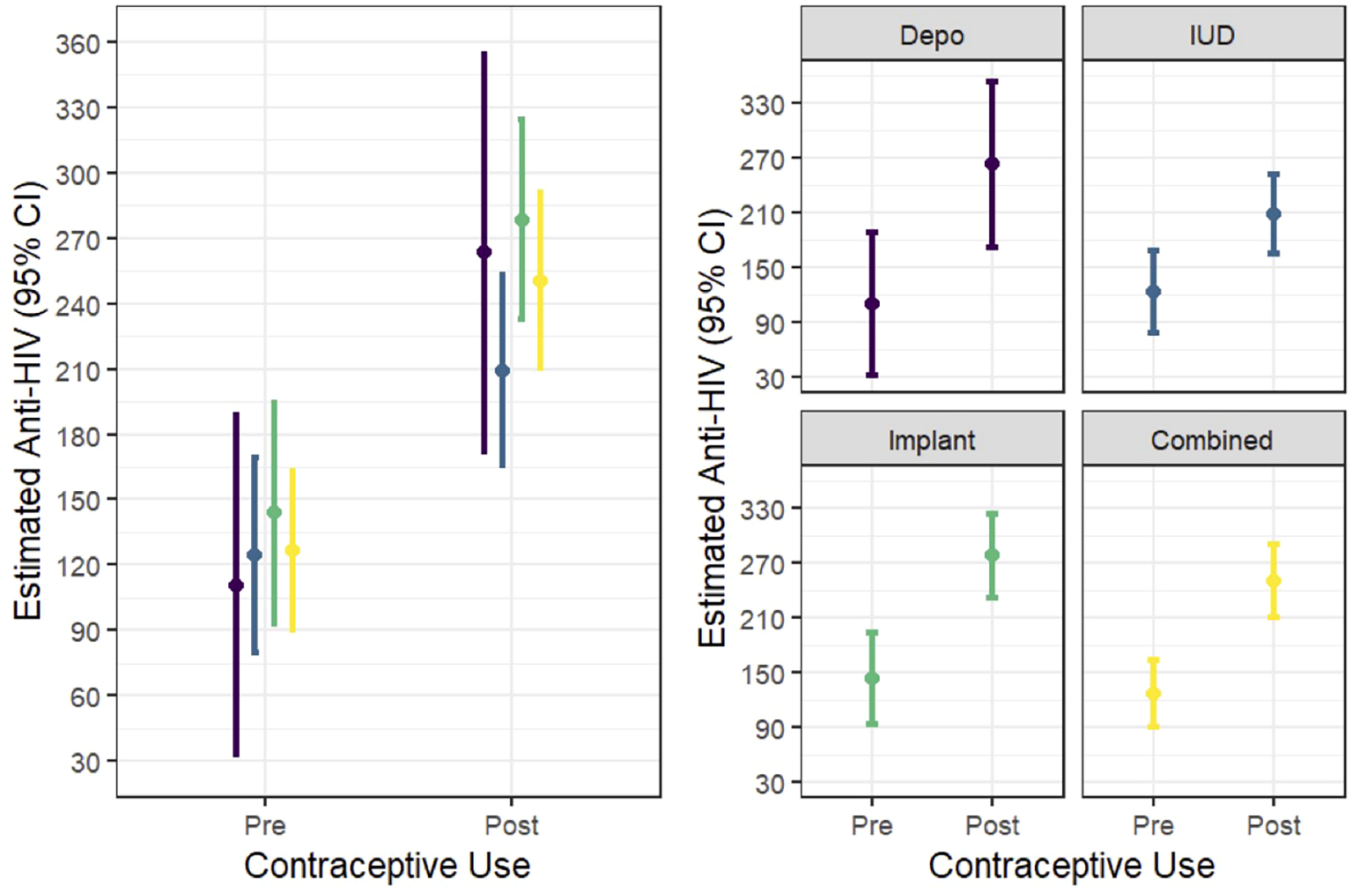
In vitro HIV activity (expressed as percent versus control) in CVL pre- and post- progestin hormonal contraception initiation.

In multivariable random effects linear modelling, percent HIV activity relative to control was higher post-HC initiation (pre-HC 126.4 (95% CI 90.1, 162.6); post-HC 250.2 (95% CI 210.0, 290.3; p<0.01; **Figure 1**) when compared to pre-HC levels. The increase was seen consistently across all three HC methods. There was no HC by time interaction for this outcome.

Vaginal microbiome characteristics were stable following HC initiation. The proportion with molecular-BV did not differ between pre-HC (0.25; 95% CI 0.16, 0.35) and post-HC visits (0.20; 95% CI 0.12, 0.30; p=0.29), and there was no interaction between HC method and time (p=0.78). Similarly, the rates of samples with <50% acid-producing *Lactobacilli* did not differ between pre-HC (0.47; 95% CI 0.33, 0.59) and post-HC visits (0.41; 95% CI 0.28, 0.56; p=0.46), and there was no interaction between HC method and time (p=0.45). We next examined whether molecular-BV or percent acid producing lactobacilli species moderated the association between HC and outcomes (cellular immune markers and HIV enhancement). There was no evidence of effect modification by molecular-BV status or *Lactobacillus* proportion for CCR5 expression in any tissue type (endocervical cells, cervical tissue, or CVL; **Supplementary Table S2**). Further, there was no evidence of effect modification for any cell type in CVL. There was evidence that molecular-BV and percent *Lactobacillus* moderated the effect of HC on proportion of CD45+ cells in the endocervical compartment and that molecular-BV or percent *Lactobacillus* moderated the relationship among several cell types in cervical tissue (CD45+, CD3+, CD4+, CD38+, and α4β7+ cells; **Supplementary Table S2**). Finally, the effect of HC on *in vitro* HIV activity was moderated by molecular-BV such that HIV enhancement was greater in the presence of molecular-BV (p=0.01) and in the presence of <50% *Lactobacillus* (p<0.01; **Supplementary Table S3**).

## Discussion

Overall, we found no substantial evidence of a change in immune cell populations that would alter HIV risk after initiation of three commonly-used progestin-based contraceptive methods: DMPA, ENG-implant, and LNG-IUD across all three contraceptive methods. However, there was an increase in the ability of genital tract secretions (CVL) to enhance HIV infection *in vitro* after HC initiation, which was consistent across the three HC modalities. The immune cell findings align with those of the ECHO trial, in which over 7,000 women were randomized to either DMPA, copper IUD, or LNG-implant (13). HIV incidence in that study was 4.9 per 100 woman-years in the DMPA group versus 3.9 and 3.3 in the copper IUD and LNG-implant groups, respectively, and there were no pairwise group differences. Our finding of baseline differences in sociodemographic variables and immune makers between women electing DMPA versus ENG-implant or LNG-IUD contraceptive methods underscores the potential for confounding to influence an observed relationship between contraceptive method and HIV acquisition, which could explain results from meta-analyses of observational studies. Our findings complement the epidemiologic evidence generated from ECHO and reassurance that the choice of contraceptive type does not pose additional HIV infection risk to users.

There have been few studies of the effects of progestin contraception on FGT cellular immunological markers or vaginal microbiome *in vivo* (23), especially for methods other than DMPA. There were no differences in density of CCR5+ T cells among 30 women initiating DMPA in Seattle and no differences in frequencies of CCR5+ T cells or activated T cells in 21 adolescent and young women using DMPA in Cape Town (24, 25). Among 24 HIV negative women in Malawi randomized to DMPA or LNG-implant, there were no changes in cytokine concentration or vaginal microbiome characteristics after initiation of contraception and up to 6 months of follow-up (26). These results are in contrast to other studies. One small study of 15 women in Virginia found higher median concentration of CCR5+ T cells in the FGT after initiation of DMPA (27), and our pilot study of 18 women initiating ENG-implant found higher percentage of CD4+ T cells expressing CCR5 in the FGT after initiation of the ENG-implant (28). A sub-study of the ECHO trial (N = 58) found higher frequency of Th17-like T cells in the endocervix of DMPA users one month after initiation (29). A larger study of 152 women using injectable progestin-only contraception (three quarters of these using DMPA and one quarter norethindrone enanthate) compared to 222 women not using hormonal contraception in South Africa found higher proportion of cytobrush-collected endocervical T cells expressed CCR5 in the injectable group (30). The authors also found higher co-expression of CD25 but no other markers of activation (HLA-DR and CD38) in the CD4+ T cells of progestin users but some imbalance in baseline characteristics of the progestin and no contraception groups should be noted. Our results could also have differed due to variation in the populations under study; in the South African study, median age was 21, *C. trachomatis* prevalence was 13%, and HIV incidence was 7.4 per 100 person-years. Indeed, effect modification, or the concept that effects of contraception on the FGT may vary across sub-groups, could also explain some of the heterogeneity in reported studies. For example, in a sub-study of the ECHO trial, authors found increased vaginal inflammatory cytokines at 1 and 3 months after initiation of all three contraceptive methods (DMPA, LNG-implant, and Copper IUD) but only among those with low vaginal inflammation at baseline (31). Women with a high baseline inflammatory milieu experienced no change or a decrease in vaginal cytokines.

In this study, we observed enhancement of *in vitro* HIV infection of target cells relative to control following HC initiation. The effects were consistent across all three types of progestin HCs and across time. Prior studies have shown that CVL may inhibit or enhance HIV infection of cells, but the clinical implications of this activity and modulating factors have not been identified (32, 33). A recent study of HIV negative pre-menopausal women found the CVL of women with Amsel-BV enhanced HIV infection *in vitro* whereas the CVL of BV negative women inhibited HIV infection (34). Consistent with that study, we found that CVL from participants with microbiologic evidence of BV exhibited greater enhancing activity after initiating HC compared to women with an optimal microbiome. Vaginal mucosal immunology is complex and presents multiple barriers against HIV infection. In light of our findings, more detailed studies that examine the wider scope of factors that influence vaginal mucosal immunity, including innate soluble activity and the factors that influence vaginal pH, are warranted.

In our analysis, molecular-BV also moderated the association between HC and several immune markers, particularly in the cervical tissue compartment, and only CD45+ cells in the endocervical compartment. However, molecular-BV did not moderate CCR5 expression in any compartment nor any cellular immune marker in the CVL. A study among serodiscordant couples in Zambia found higher risk of HIV acquisition with DMPA and oral contraceptive pill use where BV was positive, but that HC was not associated with HIV acquisition in BV negative individuals (35). Differences in the role of BV in altering the association between contraception and HIV risk could be due to differences in diagnostic methods (wet prep/gram stain in the prior study versus molecular methods utilized in CHIME) and/or variations in bacterial composition of BV, which is a heterogenous condition, in different populations and tissue types. More detailed characterization and analyses of the vaginal microbiome are outside the scope of this manuscript, but a separate presentation of these findings is planned.

Strengths of this study include longitudinal sampling with a before and after design allowing for measurement and statistical adjustment of cellular immune parameters prior to initiation of contraception, selection of contraceptive methods that are user invariant, rigorous evaluation of multiple cellular immune parameters in multiple tissue types, molecular methodology for diagnosis of BV, and robust statistical methods to adjust for potential sources of variation. Limitations of the study include imbalance in those choosing DMPA versus the other methods (though this likely reflects real-world use, and most women have contraceptive choices) and low prevalence of concurrent STIs. There are several additional T cell markers not included in this study that have been shown to be related to HIV acquisition risk and/or the HIV reservoir including CD44, CD28, CD127, and the IL-21 receptor (36). We chose to measure the HIV co-receptor as the primary study outcome, and markers associated with activation, cell trafficking, cell regulation, proliferation, and tissue residence that have known associations with HIV acquisition as secondary outcomes. Technical constraints in flow cytometry, need for efficient use of available sample material without undue burden to participants, potential redundancy in markers, and cost constraints limited the number of markers that could be included. There are other types of progestin-based contraception that were not included in this study, including norethindrone and drospirenone progestin-only pills, and our results may not generalize to other progestin-based methods. An additional study limitation is inability to achieve target sample size due to the impacts of the COVID-19 pandemic. Despite this last limitation, the repeated measures design improves our statistical power, and our sample size is in accordance with or larger than most similarly designed projects (23). Furthermore, we demonstrate that the variability of individual immune markers is high both within individuals over time and between individuals such that the effect of the contraceptive is unlikely to overpower the multifactorial determinants of the immune marker expression.

In summary, our findings are overall reassuring that use of some of the most common and effective contraceptive methods elected by women in the U.S. and globally - DMPA, ENG-implant, and LNG-IUD - do not confer immunologic changes that would increase risk of HIV acquisition, though a finding of increased *in vitro* HIV activity after HC initiation warrants further study. Given the enormous individual and societal benefits conferred by contraception to those who do not desire pregnancy, the CHIME study provides a crucial and complementary piece of evidence that these medications can be used safely.

## Data Availability

The raw data supporting the conclusions of this article will be made available by the authors, without undue reservation.
