# Supplementary figures and images for: No change in key HIV target cell markers following initiation of three progestin-based hormonal contraception methods: findings from the CHIME study
Source: Front Immunol. 2025 Nov 27;16:1655678. doi: 10.3389/fimmu.2025.1655678 (PMC12695839; doi:10.3389/fimmu.2025.1655678)

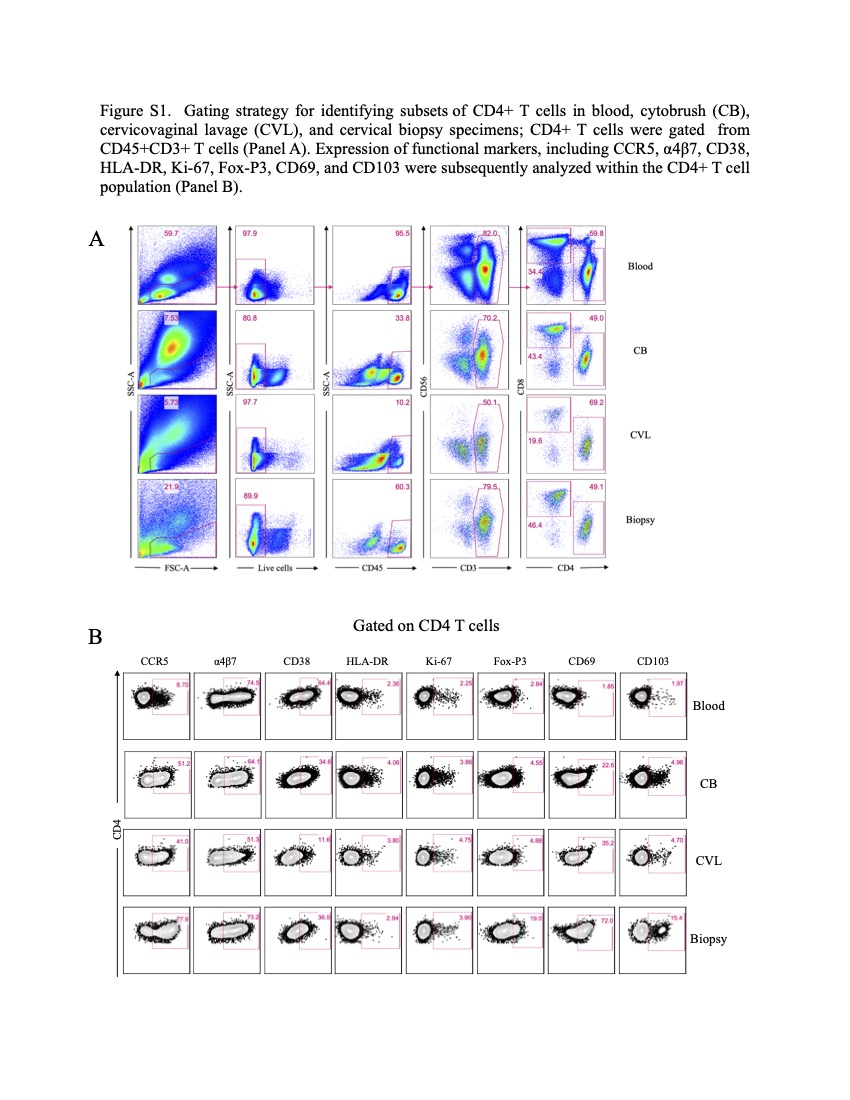

Supplement: Supplementary file 1 [file Image1.jpeg]

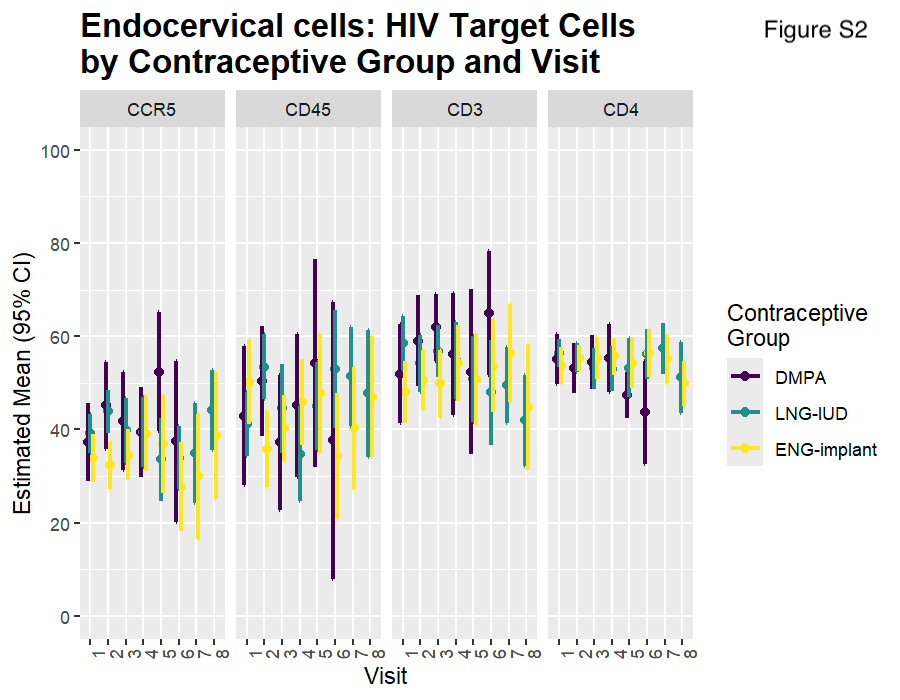

Supplement: Supplementary file 2 [file Image2.tiff]

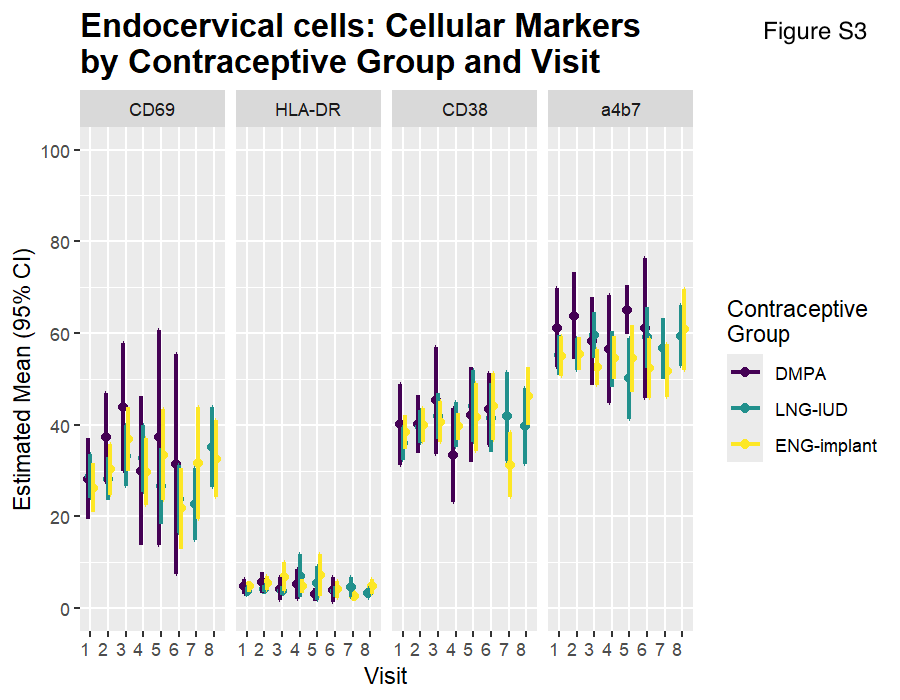

Supplement: Supplementary file 3 [file Image3.tiff]

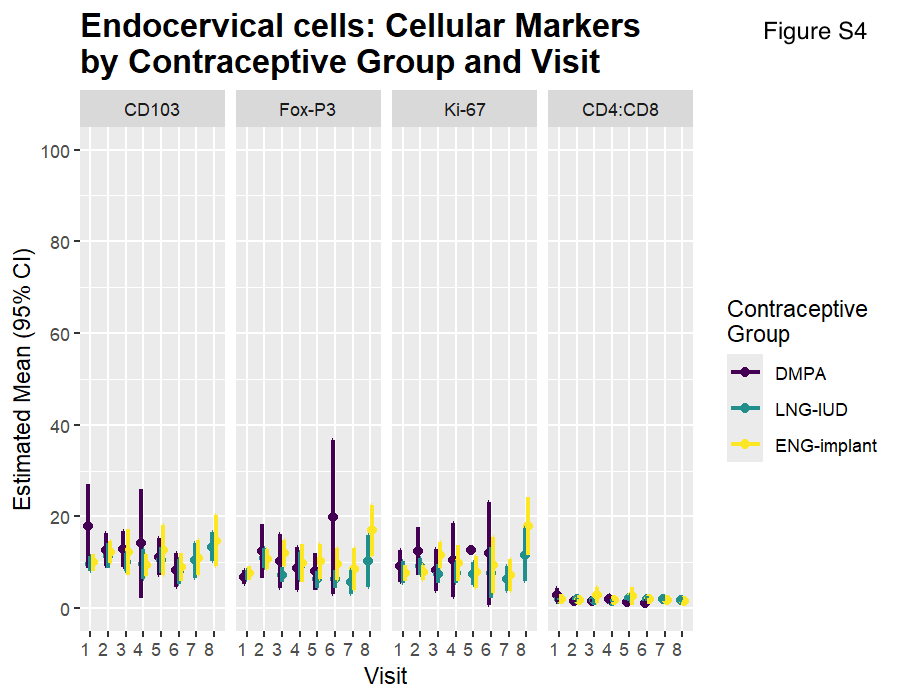

Supplement: Supplementary file 4 [file Image4.tiff]

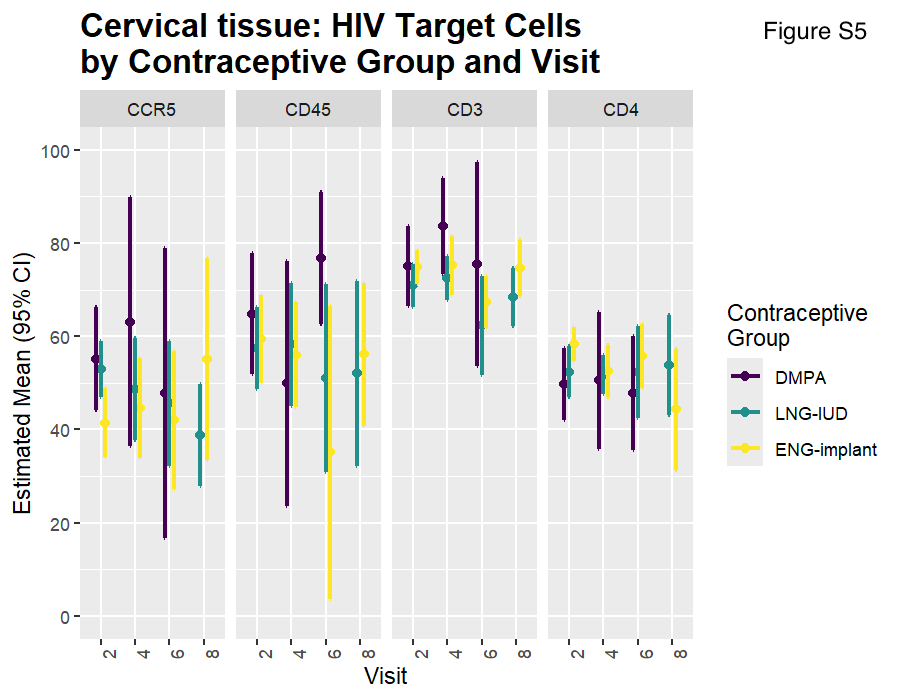

Supplement: Supplementary file 5 [file Image5.tiff]

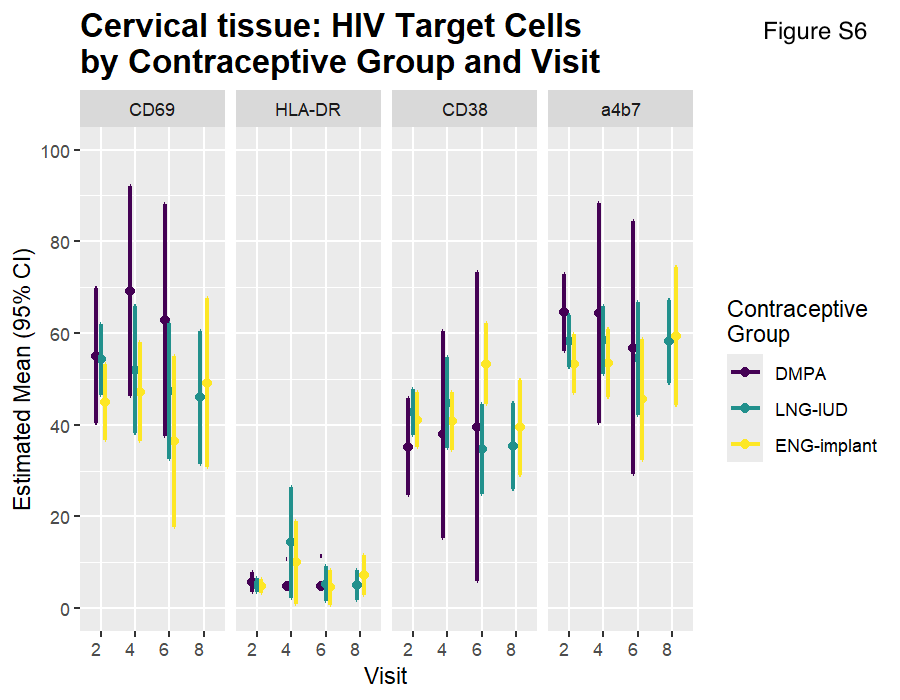

Supplement: Supplementary file 6 [file Image6.tiff]

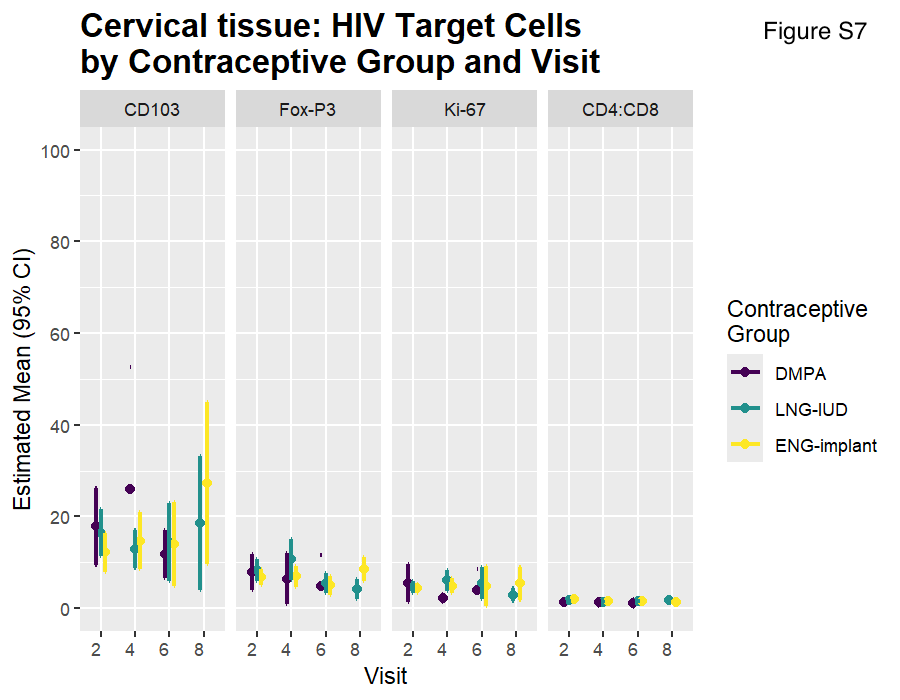

Supplement: Supplementary file 7 [file Image7.tiff]

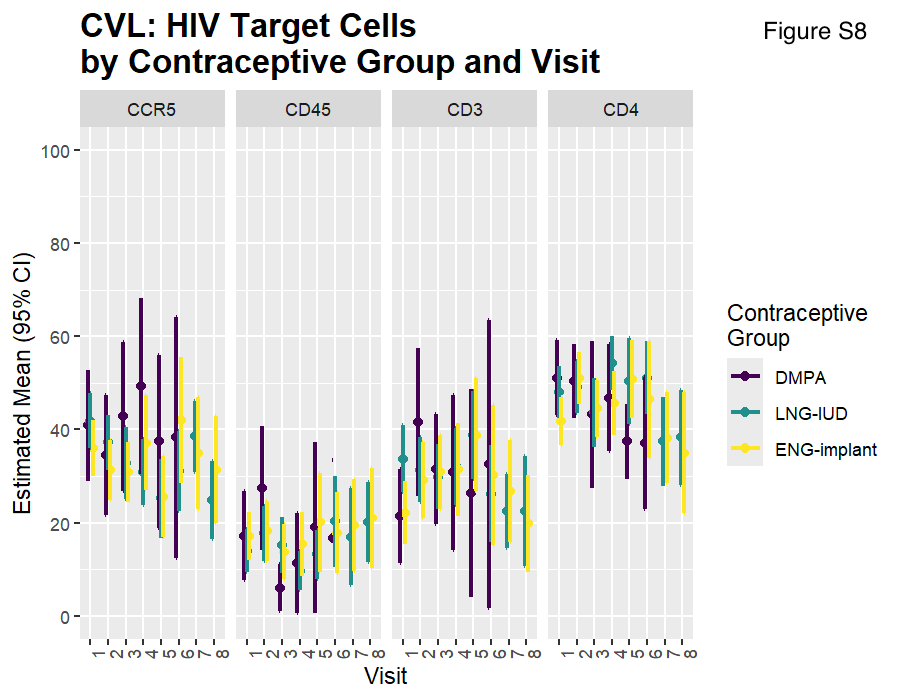

Supplement: Supplementary file 8 [file Image8.tiff]

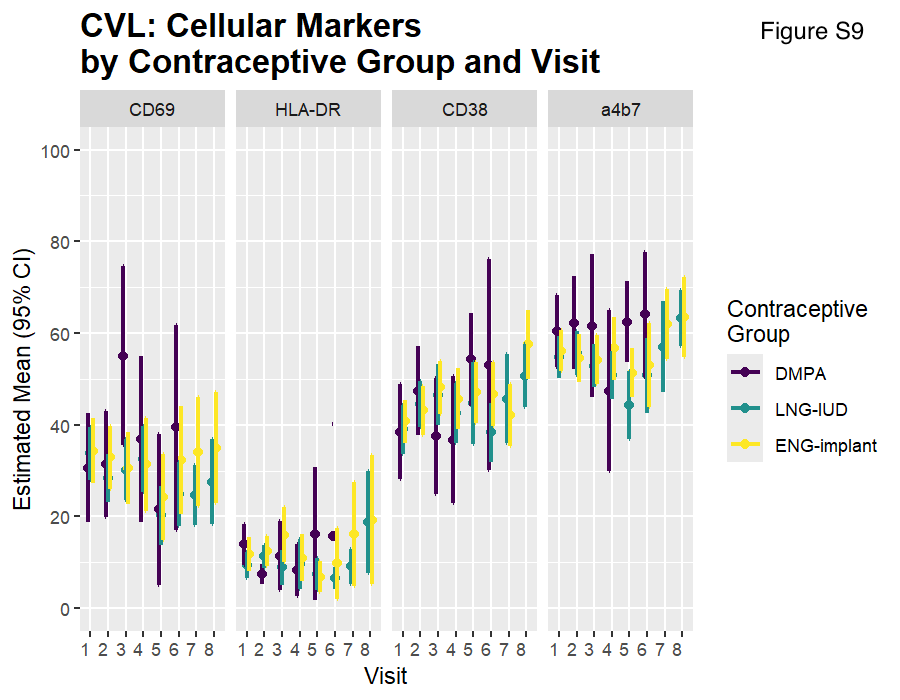

Supplement: Supplementary file 9 [file Image9.tiff]

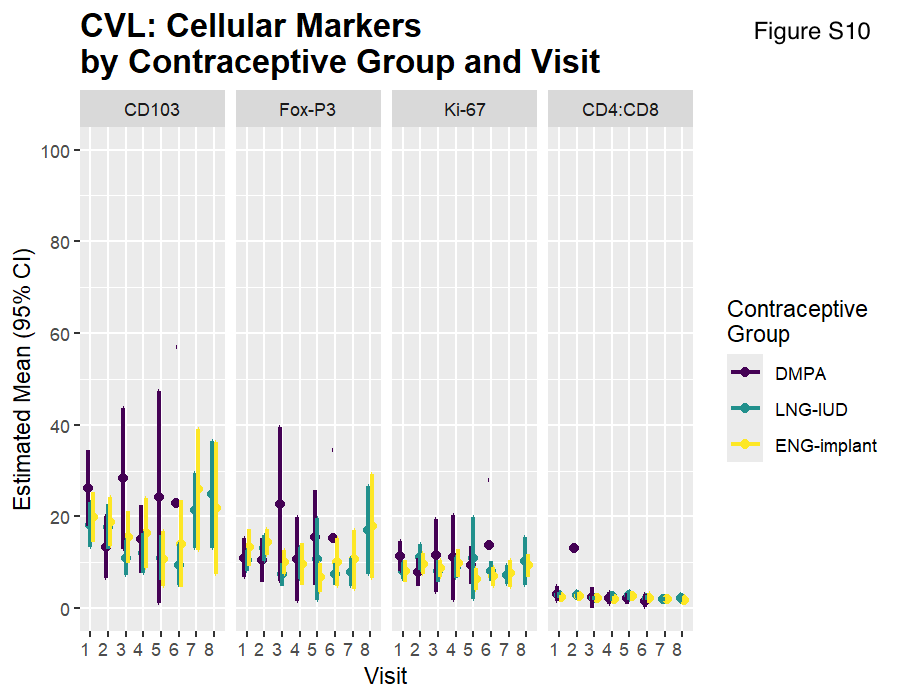

Supplement: Supplementary file 10 [file Image10.tiff]

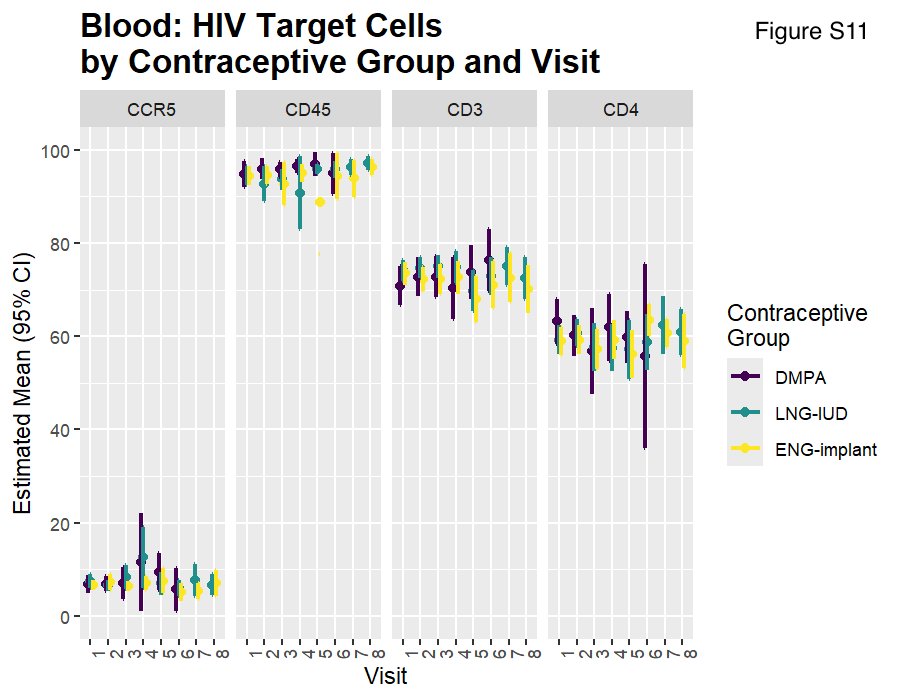

Supplement: Supplementary file 11 [file Image11.tiff]

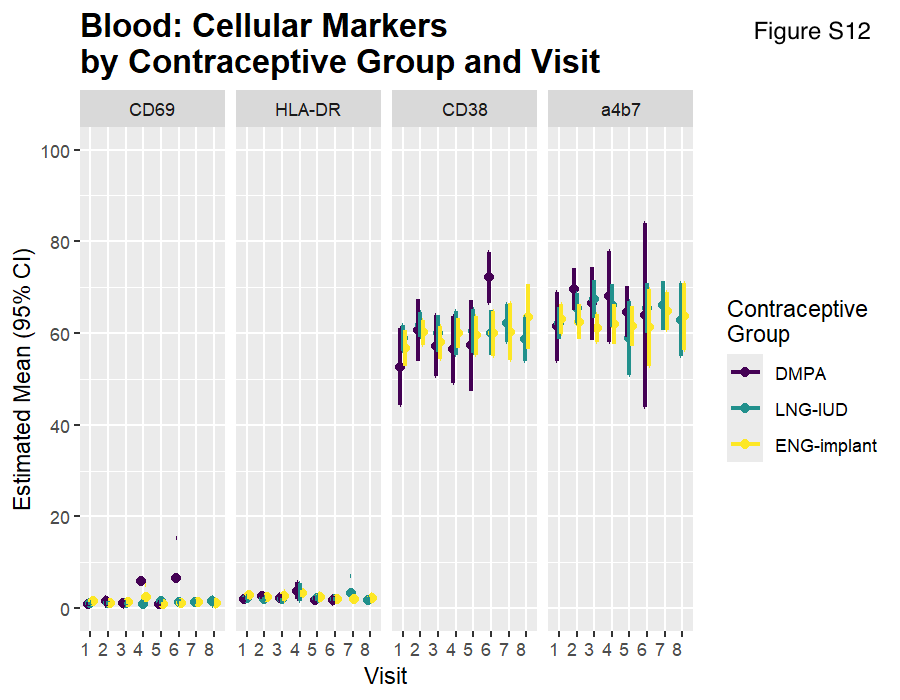

Supplement: Supplementary file 12 [file Image12.tiff]

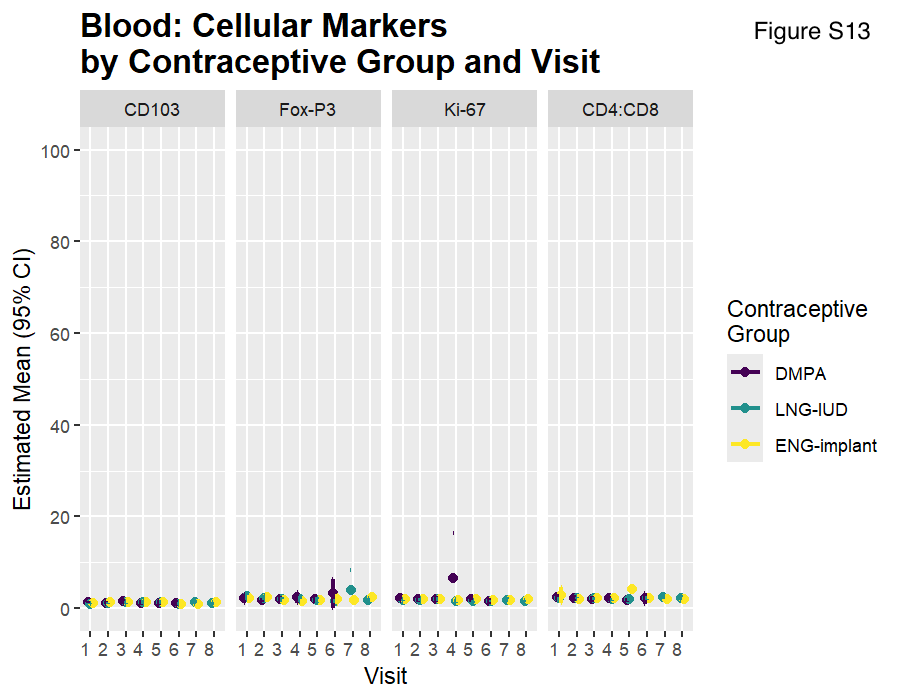

Supplement: Supplementary file 13 [file Image13.tiff]
