# Supplementary material for: No change in key HIV target cell markers following initiation of three progestin-based hormonal contraception methods: findings from the CHIME study
Source: Front Immunol. 2025 Nov 27;16:1655678. doi: 10.3389/fimmu.2025.1655678 (PMC12695839; doi:10.3389/fimmu.2025.1655678)
Supplement: Supplementary file 14 [file Table1.docx]

Table S1. Change in HIV target cells across the lower genital tract and blood of adult HIV-negative women by hormonal contraception method.

|  |  | CCR5 Pre-HC | CCR5 Post-HC |  |  |
| --- | --- | --- | --- | --- | --- |
| Tissue | HC | Estimate (95% CI) | Estimate (95% CI) | p-value* | p-value** (HC*time) |
| Endocervical cells | DMPA | 41.2 (34.7-47.7) | 45.1 (38.2-52.1) | 0.33 | 0.26 |
|  | ENG-implant | 34.3 (30.2-38.5) | 37.1 (33.2-41.1) | 0.22 |  |
|  | LNG-IUD | 42.5 (38.6-46.4) | 40.7 (36.8-44.7) | 0.43 |  |
| Cervical tissue | DMPA | 55.2 (44.5-65.9) | 57.1 (44.1-70.1) | 0.82 | 0.11 |
|  | ENG-implant | 41.3 (34.5-48.2) | 47.0 (39.8-54.2) | 0.24 |  |
|  | LNG-IUD | 53.0 (46.1-59.6) | 44.9 (38.0-51.8) | 0.08 |  |
| CVL | DMPA | 37.6 (30.2-45.1) | 44.2 (36.3-52.1) | 0.18 | **0.01** |
|  | ENG-implant | 34.9 (30.1-39.7) | 34.6 (30.1-39.1) | 0.92 |  |
|  | LNG-IUD | 40.6 (36.1-45.1) | 32.4 (27.9-36.9) | **<0.01** |  |
| Blood | DMPA | 6.84 (4.66-9.02) | 8.71 (6.47-11.0) | 0.21 | 0.27 |
|  | ENG-implant | 6.95 (5.57-8.33) | 6.53 (5/31-7.74) | 0.62 |  |
|  | LNG-IUD | 7.06 (5/77-8.35) | 8.22 (7/05-9.40) | 0.15 |  |

DMPA, depot medroxyprogesterone acetate; ENG, etonogestrel; HC, hormonal contraception; LNG-IUD, levonorgestrel intrauterine device

*Compares CCR5 pre-HC (visits 1 and 2) and post-HC (visits 3-8) for each method

**Examines the interaction between different HC methods and time.
