# Supplementary material for: No change in key HIV target cell markers following initiation of three progestin-based hormonal contraception methods: findings from the CHIME study
Source: Front Immunol. 2025 Nov 27;16:1655678. doi: 10.3389/fimmu.2025.1655678 (PMC12695839; doi:10.3389/fimmu.2025.1655678)
Supplement: Supplementary file 15 [file Table2.docx]

Table S2. Effect modifications of HC on immunologic marker by molecular-BV status and acid-producing *Lactobacillus* proportion.

|  |  |  |  |
| --- | --- | --- | --- |
| Tissue | Marker | BV by HC/visit*  p-value | % Lactobacillus by HC/visit*  p-value |
| Endocervical cells | CD45 | **<0.01** | **0.02** |
|  | CD3 | 0.47 | 0.65 |
|  | CD4 | 0.80 | 0.50 |
|  | CCR5 | 0.60 | 0.90 |
|  | CD69 | 0.25 | 0.19 |
|  | HLA-DR | 0.14 | 0.51 |
|  | CD38 | 0.84 | 0.58 |
|  | α4β7 | 0.59 | 0.16 |
|  | CD103 | 0.72 | 0.82 |
|  | Fox-P3 | 0.12 | 0.96 |
|  | Ki-67 | 0.84 | 0.10 |
|  | CD4:CD8 | 0.82 | 0.92 |
| Cervical tissue | CD45 | **0.03** | 0.19 |
|  | CD3 | 0.36 | **0.01** |
|  | CD4 | **0.02** | 0.91 |
|  | CCR5 | 0.11 | 0.96 |
|  | CD69 | 0.07 | 0.81 |
|  | HLA-DR | 0.70 | 0.36 |
|  | CD38 | **0.01** | 0.94 |
|  | α4β7 | **0.04** | 0.51 |
|  | CD103 | 0.10 | 0.93 |
|  | Fox-P3 | 0.96 | 0.22 |
|  | Ki-67 | 0.98 | 0.36 |
|  | CD4:CD8 | 0.06 | 0.94 |
| CVL | CD45 | 0.60 | 0.55 |
|  | CD3 | 0.56 | 0.44 |
|  | CD4 | 0.30 | 0.65 |
|  | CCR5 | 0.58 | 0.33 |
|  | CD69 | 0.21 | 0.50 |
|  | HLA-DR | 0.41 | 0.24 |
|  | CD38 | 0.07 | 0.17 |
|  | α4β7 | 0.65 | 0.86 |
|  | CD103 | 0.35 | 1.0 |
|  | Fox-P3 | 0.80 | 0.97 |
|  | Ki-67 | 0.57 | 0.43 |
|  | CD4:CD8 | 0.94 | 0.81 |

BV, bacterial vaginosis; CVL, cervicovaginal lavage; HC, hormonal contraception

*HC/visit includes the following categories: pre-HC, post-HC DMPA, post-HC LNG-IUD, post-HC ENG implant (see methods section).
