# Supplementary material for: No change in key HIV target cell markers following initiation of three progestin-based hormonal contraception methods: findings from the CHIME study
Source: Front Immunol. 2025 Nov 27;16:1655678. doi: 10.3389/fimmu.2025.1655678 (PMC12695839; doi:10.3389/fimmu.2025.1655678)
Supplement: Supplementary file 16 [file Table3.docx]

Table S3. Change in *in vitro* HIV activity (expressed as percent versus control) pre-and post- progestin hormonal contraception initiation and by vaginal microbiome status.

|  | Pre-HC^¶^ | Post-HC^†^ | p-value* |
| --- | --- | --- | --- |
|  | Estimate (95% CI) | Estimate (95% CI) |  |
| **Molecular-BV status (CST-IV vs other CST)** | | | |
| BV | 114.6 (65.4, 163.8) | 279.8 (228.8, 330.8) | **0.01** |
| No BV | 133.4 (97.8, 169.0) | 208.0 (173.0, 243.0) |  |
| **% Acid-producing *Lactobacillus* (excludes *L. iners)*** | | | |
| <50% *Lactobacillus* | 118.4 (78.4, 158.4) | 261.0 (221.4, 300.6) |  |
| ≥50% *Lactobacillus* | 142.7 (101.4, 183.9) | 187.9 (145.9, 229.8) | **<0.01** |

BV, bacterial vaginosis; CST, community state type; HC, hormonal contraception

¶Includes visits 1 and 2 (see Methods)

†Includes visits 3-8 (see Methods)

*Examines the interaction between microbiome status and HC/visit on anti-HIV activity
